# Genetic Association between Amyotrophic Lateral Sclerosis and Cancer

**DOI:** 10.3390/genes8100243

**Published:** 2017-09-27

**Authors:** Y-h. Taguchi, Hsiuying Wang

**Affiliations:** 1 Department of Physics, Chuo University, 1-13-27 Kasuga, Bunky-ku, Tokyo 112-8551, Japan; tag@granular.com; 2 Institute of Statistics, National Chiao Tung University, Hsinchu 30010, Taiwan

**Keywords:** amyotrophic lateral sclerosis, biomarker, cancer, gene, microarray

## Abstract

Amyotrophic lateral sclerosis (ALS) is a fatal neurodegenerative disease. An ALS drug, Riluzole, has been shown to induce two different anticancer effects on hepatocellular carcinoma (HCC). In light of this finding, we explore the relationship between ALS and cancer, especially for HCC, from the molecular biological viewpoint. We establish biomarkers that can discriminate between ALS patients and healthy controls. A principal component analysis (PCA) based unsupervised feature extraction (FE) is used to find gene biomarkers of ALS based on microarray gene expression data. Based on this method, 101 probes were selected as biomarkers for ALS with 95% high accuracy to discriminate between ALS patients and controls. Most of the genes corresponding to these probes are shown to be related to various cancers. These findings might provide a new insight for developing new therapeutic options or drugs for both ALS and cancer.

## 1. Introduction

Amyotrophic lateral sclerosis (ALS) is a fatal neurodegenerative disease, which is caused by degeneration of upper motor neurons of the motor cortex and corticospinal tract and lower motor neurons of the brainstem and spinal cord. Eventually, it can affect the ability to control the muscles for movement, leading to death. Despite many studies to investigate this disease, no effective therapeutics have been identified to prevent or stop neuronal death in patients so far. Most of the cases are caused by unknown reasons. Only 5–10% of ALS cases have a family history of the disease [1]. Familial cases have been linked to mutations in a number of genes, including Cu/Zn superoxide dismutase 1 (SOD1), TAR DNA binding protein (TARDBP), fused in sarcoma (FUS), and chromosome 9 open reading frame 72 repeat expansions (C9ORF72) [2,3].

The comorbidity of ALS with other disorders has been discussed in previous cohort studies. Increasing evidence suggests that ALS is inversely related to cancer [4]. An increased risk of ALS was observed during the first year after cancer diagnosis, and in contrast, a lower risk of cancer was observed in ALS patients after diagnosis compared with ALS-free individuals [5]. Most discussions of the comorbidity of ALS with cancer are based on cohort studies, case reports of individual observations, or sequence mutation analysis. Genetic speculation through the microarray data with respect to the relationship between ALS and cancer has not been much explored in the literature. In this study, we aim to explore the potential genetic link between ALS and cancer, especially for hepatocellular carcinoma (HCC) and pancreatic adenocarcinoma (PAAD), which are primary tumors of the liver and pancreas.

The clinical evidence of the association between ALS and cancer in the literature includes the effect of ALS drugs in cancer therapy and the association between paraneoplastic antibodies and ALS. An ALS drug, Riluzole, was shown to induce two different anti-cancer effects on HCC [5]. A male patient was reported with C9ORF72 familial ALS and paraneoplastic antibodies (anti-Ma2/Ta) [6]. Manganese superoxide dismutase (MnSOD) can be induced to protect against pro-oxidant insults resulting from certain tumors and ALS [7]. Reports have shown increased MnSOD protein or activity in ALS patient samples [8,9]. However, the role of MnSOD in cancer is still disputable as some studies have reported that tumor cells/tissues contain decreased MnSOD activity whereas some describe an increase of MnSOD protein in tumor cells/tissues [10,11,12].

To find the biological significance of the association between ALS and cancer, we analyze microarray gene expression data for ALS and healthy control samples. All samples were from human biopsies, either from healthy muscles or from the muscle of patients who were clearly diagnosed as having ALS [13]. The findings in this study enhance the biological evidence connecting ALS with cancer and might provide a new insight for developing new therapeutic options for both diseases, and a new perspective for understanding the pathophysiology of ALS.

## 2. Materials and Methods

In order to clarify the relationship between ALS and cancers from the molecular biological viewpoint, we first establish gene biomarkers that can discriminate between ALS patients and healthy controls, and then we investigate whether these biomarkers are functional/dysfunctional in a cancer-specific manner. For this purpose, we applied the principal component analysis (PCA) based unsupervised feature extraction (FE) [14,15] to gene microarray expression profiles E-MEXP-3260 [13] to discover potential ALS probes.

Gene expression profiles used in this study were downloaded from ArrayExpress using the accession number E-MEXP-3260. It includes 10 normal samples (01a to 10a) and nine ALS samples (11a to 19a). The 10 normal samples include five females and five males; the nine ALS samples are all males. The ALS diagnosis of the nine patients with probable or definite ALS is based on the revised El Escorial criteria [16] by performing an open biopsy in the middle portion of deltoid muscle on these patients [13]. All patients had sporadic ALS, and presented with symptoms of limb onset. All of them underwent a complete needle electromyography investigation. The normal control samples were obtained from 10 subjects without any significant neurological history, when undergoing a shoulder orthopedic surgery. The samples were processed and stored using standard procedures [17]. The gender and age of the samples can be found in Table 1 of [13]. The average age of the 10 normal samples and the nine ALS samples are 52.1 ± 7.8 and 48.5 ± 15.9 years, respectively. The time from onset of symptoms to biopsy, the revised ALS functional rating scale and other information of the nine patients can be obtained from that table.

To analyze the data, the obtained 19 CHP format files, which contains probe set analysis results generated from Affymetrix software, were loaded into R (R Core Team, 2015) using the ‘readChp’ function in the package ‘affxparser’. Then, the component ‘QuantificationEntries$Signal’ of the output of ‘readChp’ was used for further analyses. Gene expression profiles were normalized with each sample (i.e., each column was normalized).

Suppose $x_{ij}$ is the gene expression of the ith gene in the jth sample. In PCA based unsupervised FE, not samples but genes are embedded into low dimensional space. Thus, principal component (PC) scores attributed to genes are computed as a component of eigenvector, $u_{k}$, of $XX^{T}$, where X is the matrix of $x_{ij}$ and $X^{T}$ is its transposed matrix

$$XX^{T}u_{k}=\lambda_{k}u_{k},$$

where $\lambda_{k}$ is the eigenvalues ($\lambda_{k+1}<\lambda_{k}$). Principal component loading attributed to samples are the components of the vector $v_{k}=X^{T}u_{k}$ that is the eigenvector of $X^{T}X$ because

$$X^{T}Xv_{k}=X^{T}XX^{T}u_{k}=X^{T}\lambda_{k}u_{k}=\lambda_{k}X^{T}u_{k}=\lambda_{k}v_{k}.$$

Since we could identify that the second PC loading is distinct between healthy controls and ALS patients with a *p*-value 9.4 × 10^−7^ by the Student’s *t*-test, we decided to select genes with the second PC score. *p*-Values were attributed to genes with assuming that the test statistic ${\left(u_{2i}/\sigma_{2}\right)}^{2}$ follows a $\chi^{2}$ distribution where $u_{2i}$ is the ith component of $u_{2}$ and $\sigma_{2}$ is the standard deviation of $u_{2i}$. *p*-Values were further adjusted by Benjamini and Hochberg criterion [18] and genes associated with adjusted *p*-values less than 0.01 were selected.

In order to examine whether the selected probes can discriminate between ALS patients and healthy controls, PC loading was recomputed using only the selected probes and was attributed to 19 samples. Then samples were discriminated using linear discriminate analysis (LDA) with the second PC loading. Leave one out cross-validation was employed.

Then we identified the genes which are associated with the selected probes by the Database for Annotation, Visualization, and Integrated Discovery (DAVID) gene ID converter [19]. Probe ID (GENBANK accession) was converted to gene symbol. Single GENBANK accession was often converted to multiple gene symbols. The obtained genes symbols were uploaded to OncoLnc [20] one by one, and cancers associated with false discovery rate (FDR) of less than 0.05 were identified as cancers associated with significant survival probabilities for the investigated genes.

All of the statistical analyses were performed using R codes. Gene expression profiles were normalized by the function ‘scale’ and PCA was performed by the function ‘prcomp’*.* Student’s *t*-test was performed by the function ‘t.test’ and LDA was performed by the function ‘lda’ in R package ‘MASS’*.*

## 3. Results

**Figure 1:**
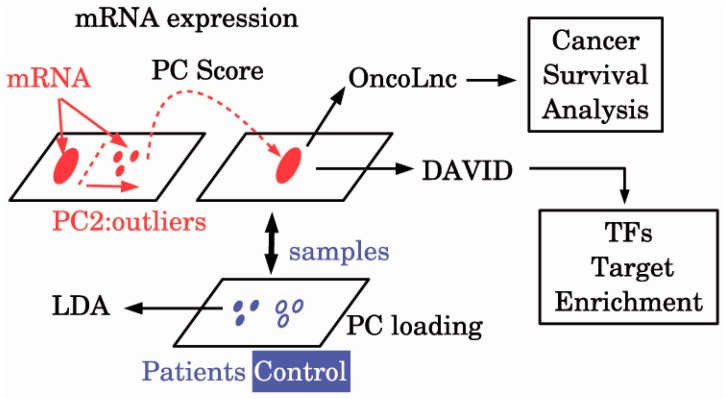
The diagram of the analysis. DAVID: Database for Annotation, Visualization, and Integrated Discovery; LDA: Linear discriminate analysis; mRNA: Messenger RNA; PC: Principal component; TFs: Transcription factors.

A schematic diagram of the analyses is provided in Figure 1. Then, 101 probes in E-MEXP-3260 were selected (Supplementary Table S1). Using these 101 probes, we can discriminate between ALS patients and healthy controls with the accuracy of 95% (Table 1) by applying the LDA. Thus, we could successfully identify biomarkers that can discriminate between ALS patients and healthy controls with high accuracy.

**Figure 2:**
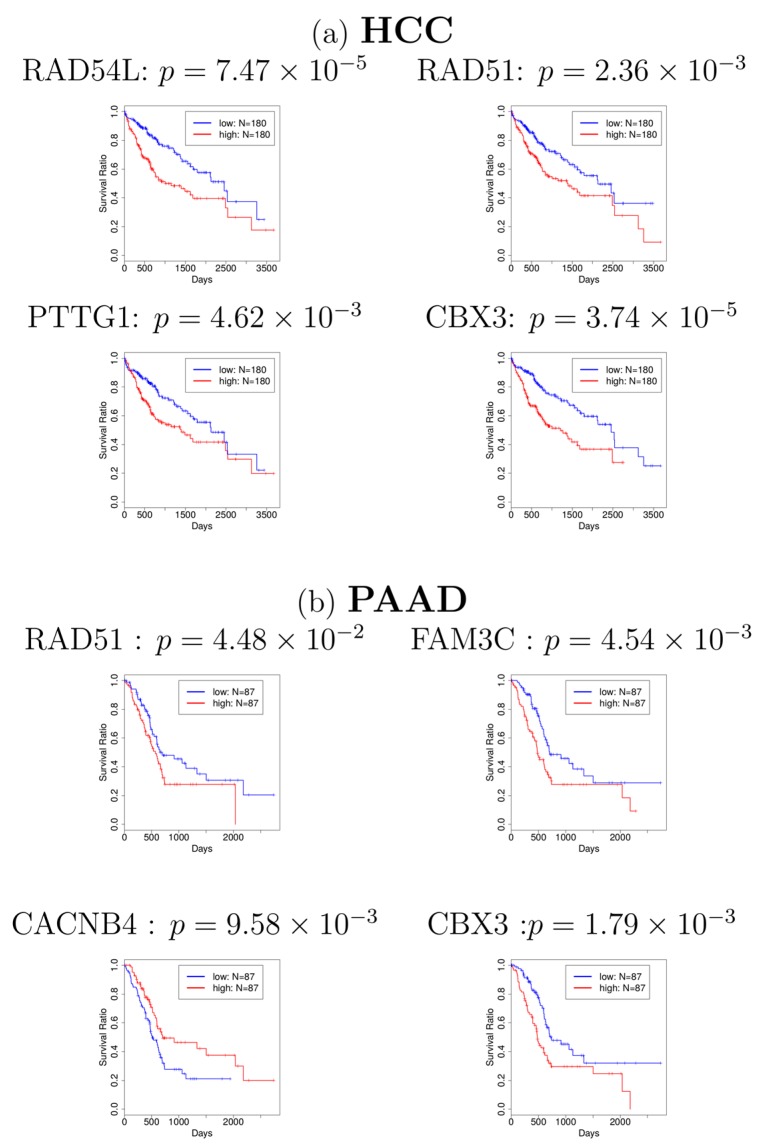
Kaplan–Meier plots for (**a**) hepatocellular carcinoma (HCC) and (**b**) pancreatic adenocarcinoma (PAAD). Blue: patients with lower gene expression (lower half, *N* = 180 for HCC, *N* = 87 for PAAD). Red: patients with higher gene expression (upper half, *N* = 180 for HCC, *N* = 87 for PAAD). Most of the figures show that patients with lower gene expression (blue line) have a higher survival probability.

Next, in order to investigate whether the genes associated with these 101 probes have a significant relationship with cancer, we first identified the genes which are associated with 101 probes. The identified genes are shown in Supplementary Table S2. Many of them have been reported to be related to cancers. For example, RAD51 was reported to be related to HCC and PAAD [21,22]. CBX3, also known as HP1γ, was also reported to be related to HCC [23] and PAAD [24]. In addition to these findings from literature review, to investigate whether most of the genes in Supplementary Table S2 are related to cancers, we adopted the database OncoLnc [20] to find the association between these genes and cancers, which is a pre-computed database for multiple cancers and has been used in calculating survival probability in several studies [25,26]. The survival analyses for genes in Supplementary Table S2 are provided in Supplementary Table S3, which shows that most of the genes identified are related to various cancers. Especially, four genes (RAD54L, RAD51, PTTG1, and CBX3) and four genes (RAD51, FAM3C, CACNB4, and CBX3) are significantly related to HCC and PAAD in the survival analyses (see Figure 2), respectively, which corresponds with some of the previous studies. Finally, we selected 68 genes (Supplementary Table S4) that turn out to be significantly associated with cancer survival probability. To our knowledge, this is the first report that relates gene expression profiles of ALS to cancer survival analysis

## 4. Discussion

Although we could successfully identify ALS biomarkers that are related to cancer from the survival analyses, more detailed biological consideration of these biomarker genes might provide an understanding of the biological background shared between ALS and cancer. In order to clarify this point, we uploaded the 68 genes (Supplementary Table S4) to DAVID to perform pathway enrichment analysis. Multiple Reactome pathway enrichment was observed (Supplementary Table S5), which included many terms associated with cancer. For example, the terms, R-HSA-975956 and R-HSA-975957, were included in the nonsense-mediated decay (NMD) pathway, and NMD was known to be related to cancers. Wang et al. [27] reported that inhibition of NMD by the tumor microenvironment promotes tumorigenesis. Ionov et al. [28] found that manipulation of NMD identifies gene mutations in colon cancer cells with microsatellite instability. Nonsense-mediated decay was also suggested to play critical roles in implications for tumorigenesis [29]. On the other hand, NMD is known to block antisense oligonucleotides (ASOs), and blocking ASOs could reduce SOD1 in the central nervous system [30]. SOD1 is the famous ALS-causing gene. Li et al. [31] showed that endoplasmic reticulum (ER) stress compounded TAR DNA-binding protein 43 (TDP-43) depletion in the upregulation of NMD isoforms that had been implicated in the pathogenic mechanisms of ALS. TDP-43-associated cryptic exons was also reported to lead to NMD [32]. TDP-43 is the main component of ubiquitinated protein aggregates found in sporadic ALS patients, not only familial ALS [33]. In addition to this, expansion of hexanucleotide repeats in C9ORF72 increases the susceptibility for pathological alteration of TDP-34 [34]. The worldwide frequency of the C9ORF72 repeat expansion is now estimated to more than 30% for familial ALS, which represents more than 5% of the sporadic ALS cases [35]. Thus, these findings suggested that ALS is related to cancers. Second, signal recognition particle (SRP) related term R-HSA-1799339 in Supplementary Table S5, was enriched. Signal recognition particle is suggested to be related to cancer histology [36] and the SRP protein is also detected in colon cancer [37]. On the other hand, myopathy associated with anti-signal recognition particle (anti-SRP) is a severe necrotizing immune-mediated disease [38]. Anti-SRP myopathy also differs from other immune-mediated myopathies by its characteristically poor responsiveness to steroid monotherapy and conventional immunosuppressive therapies [39]. Moreover, there it has been reported that the enhancement of nerve inflammation causes nerve cell death [40]. As a result, SRP can associate ALS with cancers.

More interestingly, in some cases, NMD and SRP were detected together in cancer tissues. Simões et al. [41] had identified the enrichment of “SRP-dependent cotranslational protein targeting to membrane” and “nonsense-mediated decay” in the MCF-7 cell line by investigating an interactome of endogenous and overexpressed ER in MCF-7 cell line generated from breast cancer. Durmaz et al. [42] identified enrichment of “SRP-dependent cotranslational protein targeting to membrane” and “nonsense-mediated decay” infrequent subgraph mining of personalized signaling pathway networks analysis applied to breast cancer as well as glioblastoma multiforme patients. This strengthens the feasibility that NMD and SRP were identified together in the present research when cancer-related genes were sought among those related to ALS.

In addition to pathway enrichments, multiple transcription factor (TF) target genes were enriched (Supplementary Table S5). These TFs were P300, E47, AHR, HMX1, AREB6, GATA2, and MEIS1AHOXA9. As for the relationship between these TFs and ALS, E47, also known as TCF3, was reported as one of 23 candidate causal master regulators of neurodegeneration in an in vitro model of ALS [43]. Butovsky et al. [44] found that AHR was significantly upregulated in Ly6Chi monocytes in SOD1 mice. AREB6, also known as ZEB1, was suggested to be included in a regulatory circuit related to ALS pathogenesis [45]. P300, also known as EP300, also has a role in the etiology of ALS [46]. Thus, not all but the majority of these TFs were reported to be related to ALS. On the other hand, as for the relationship between cancers and these TFs, mutations truncating EP300 acetylase was reported in human cancers [47]. ZEB1 turns into a transcriptional activator by interacting with YAP1 in aggressive cancer types [48]. AHR is also known to be deeply related to cancer biogenesis [49]. Breast cancer growth and initiation were controlled by TCF3 [50]. GATA is also a well- known cancer-related TF [51]. Therefore, all of the discussions here suggested that genes that can distinguish between ALS and healthy controls can biologically bridge between ALS and cancer biogenesis.

## 5. Conclusions

In this paper, we have identified genes whose expression profiles can discriminate between ALS patients and healthy controls. Most of these gene expression profiles turned out to be related to various cancers using survival analysis, pathway enrichment analysis, and TF enrichment analysis. Furthermore, biological consideration of these genes can help us to understand the molecular biological background that ALS patients can have distinct cancer pathogenesis from other patients. Specifically, NMD and SRP, as well as enhanced TF binding protein genes, are promising candidates that relate to ALS and cancer. These findings suggested that our approach is useful for understanding the relationship between cancers and ALS, especially from a molecular biology point of view

## Figures and Tables

**Table 1:** Confusion table of LDA between amyotrophic lateral sclerosis (ALS) and normal control.

		True	
		Normal	ALS
Prediction	Normal	10	1
	ALS	0	8

## Supplementary Materials

The following are available online at www.mdpi.com/2073-4425/8/10/243/s1. Table S1: 101 probes identified by PCA based unsupervised FE, Table S2: Gene symbols associated with 101 probes identified by DAVID gene ID converter, Table S3: Cancer survival probabilities identified by OncoLnc for genes in Table S2, Table S4: Genes associated with significant cancer survival probabilities, Table S5: REACTOME and TFBS enrichment by DAVID for genes in Table S4.
